# Alpha-Gal Syndrome in the Heartland: Dietary Restrictions, Public Awareness, and Systemic Barriers in Rural Kansas

**DOI:** 10.3390/nu17193043

**Published:** 2025-09-24

**Authors:** Judith Sempa, Priscilla Brenes, Alexandra Tegeler, Jordan Looper, Michael Chao, Yoonseong Park

**Affiliations:** 1 School of Health Sciences, Kansas State University, Manhattan, KS 66506, USA; jsempa@ksu.edu; 2 Department of Animal Sciences and Industry, Kansas State University, Manhattan, KS 66506, USA; aptegeler@ksu.edu; 3 Department of Animal Science, Texas A&M University, College Station, TX 77840, USA; jtlooper@tamu.edu (J.L.); michael.chao@ag.tamu.edu (M.C.); 4 Department of Entomology, Kansas State University, Manhattan, KS 66506, USA; ypark@ksu.edu

**Keywords:** alpha-gal syndrome, tick-borne allergy, rural healthcare, dietary restrictions

## Abstract

**Background/Objectives**: Alpha-gal syndrome (AGS) is a tick-borne allergic condition increasingly prevalent in the Southern, Eastern and Mid-western regions of the United States. This study aims to assess the awareness and understanding of AGS among Kansas residents, focusing on its symptoms, impact, and available management resources. **Methods**: Two anonymous, self-reported surveys were administered using Qualtrics software [Qualtrics XM, Provo, UT, USA], one targeting Kansas Extension professionals and the other directed at Kansas community residents. **Results:** Participants from both groups demonstrated general awareness of AGS, with 82 respondents self-identifying as having the condition. Beyond the dietary restrictions associated with the condition, individuals with AGS also faced a range of social, financial and health-related challenges. The study revealed critical gaps in healthcare preparedness and support infrastructure for managing AGS in rural Kansas communities. **Conclusions**: While awareness of AGS is increasing in Kansas, individuals affected by this condition continue to encounter significant challenges. These are not limited to personal and emotional hardships but also include systemic challenges in healthcare and community support. Study findings highlight a critical gap between growing awareness and implementation of effective action.

## 1. Introduction

Alpha-gal syndrome (AGS), also referred to as “red meat allergy,” is a tick-borne allergic reaction to galactose-alpha-1,3-galactose (alpha-gal), an oligosaccharide present in most non-primate mammalian tissues, cells and fluids. The lone star tick (*Amblyomma americanum*) is the primary tick species linked to AGS and is commonly found in the Southeastern and Mid-western regions of the United States, where up to 20% of the population may carry high levels of alpha-gal antibody immunoglobulin E (IgE) [1]. When the tick bites, it introduces alpha-gal into the bloodstream, prompting the immune system to generate specific IgE antibodies against it, although the full AGS sensitization mechanism is not yet fully understood [2,3]. In AGS, allergic reactions are delayed, typically occurring 2 to 6 h after consuming mammalian meats such as pork, lamb, beef, and organ meats, and certain medications made from mammalian-based ingredients. Reactions may also be triggered by dairy products, gelatin, or even non-mammalian items that contain alpha-gal, such as carrageenan (a seaweed-derived additive). Symptoms can vary from mild manifestations, including hives, skin rashes, and swelling of the lips, tongue, throat, and eyelids, to more serious effects like wheezing, heartburn, gastrointestinal discomfort (e.g., abdominal pain, nausea, vomiting, and diarrhea), and, in severe cases, anaphylaxis [2,3,4]. AGS was first recognized in 2009 following reports of delayed anaphylaxis to red meat and reactions to the cancer drug cetuximab [5,6,7,8]. AGS has also been reported globally in the Middle East, South America, Europe, Africa, East and South Asia, Australia and New Zealand, but with different tick species as culprits [9,10,11,12,13].

Individuals residing in rural areas, like in rural Kansas, and those who spend significant time outdoors, such as foresters, hunters, hikers, and campers, are at greater risk of exposure to ticks and consequently more likely to develop AGS [14]. In the United States, between 2011 and 2019, the number of diagnosed AGS cases increased dramatically from 2330 to over 34,000, underscoring its growing prominence as a public health concern [15]. With the expansion of lone star tick populations, driven by climate variability and the rising white-tailed deer population, the incidence of AGS is expected to continue to rise [16,17].

The main recommendation for individuals recently diagnosed with AGS is to entirely avoid the meat of mammals, including beef, pork, venison, bison, buffalo, rabbit, horse, goat, and lamb [18]. Fatty meats are more consistently linked to symptoms and more intense reactions [19,20]. Mammal organ meat such as liver, kidneys, and intestines can trigger reactions and should be avoided as well. In addition, meat broth, fatback or bacon in salads, bouillon, stock and gravy should also be avoided [19,21]. According to the Center for Disease Control and Prevention (CDC) [22], non-food products to avoid include gelatin (made from collagen in pig or cow bones), lanolin, collagen, glycerin, magnesium stearate, and bovine extract (which can be used to manufacture bioprosthetic valves). Gelatin-containing medications (vaccines) and foods containing gelatin such as candies, desserts, gummy bears, and marshmallows may also cause issues. Dairy products contain small amounts of alpha-gal, especially ice cream and cream cheese which are high in fat [23,24].

AGS is often underdiagnosed and misdiagnosed due to its recent discovery, atypical presentation, and limited awareness among healthcare professionals. As concern around AGS grows, research consistently shows that healthcare providers remain largely unaware of the condition, forcing many patients to seek information from nontraditional sources [25]. This lack of health provider knowledge creates significant informational gaps that can delay diagnosis and hinder effective management. This underscores the need for more effective, community-driven strategies for disseminating health information, bridging informational gaps and ensuring timely, accurate health information, particularly in rural areas such as Kansas. Kansas, which is located in the midwestern part of the United States, is home to many rural communities where the prevalence and impact of AGS remain largely unknown. The Kansas population faces unique challenges related to its geographic location, limited specialist access, and limited healthcare communication. This study aims to investigate Kansans’ awareness and understanding of AGS, its symptoms, and available management resources. It also seeks to examine the geographic distribution of AGS, its impact, and challenges related to long-term symptom management within rural Kansas Communities. By evaluating the accessibility and effectiveness of current educational and informational resources, this study seeks to uncover critical gaps in public health outreach. The findings will inform targeted strategies for prevention, education and ongoing support to empower both healthcare providers and community members to recognize and respond to AGS more effectively.

## 2. Materials and Methods

This study followed the STROBE statement guidelines for cross-sectional studies and the CHERRIES checklist for internet E-surveys [26,27]. It involved two anonymous surveys of Kansas Extension professionals and Kansas community residents, administered using Qualtrics software [Qualtrics XM, Provo, UT, USA]. The research received approval from the Kansas State University Institutional Review Board [IRB-12256]. Participants were notified that their consent was implied upon starting the survey. A $25 gift card was offered as an incentive upon completion of an additional form following the initial survey.

### 2.1. Study Participants

Eligible participants included Kansas Extension professionals and residents of Kansas who were over the age of 18. Residency was confirmed by filtering out responses with IP addresses originating outside of Kansas.

### 2.2. Survey Development

Both survey instruments gathered data on participants’ age, gender, race, ethnicity, occupation, and county of residence or work. They included both closed and open-ended questions across several topics. These included awareness and knowledge of AGS, whether participants had heard of the condition, were familiar with its symptoms and knew of its link to tick bites. Participants were also asked how many individuals they knew had been affected by AGS in their county. Additional questions explored how AGS impacted affected individuals’ diets and daily lives, and public perceptions regarding the availability of AGS information and whether respondents believed more resources were needed.

The community survey instrument also included questions on environmental and behavioral risk factors where respondents were probed about their current occupation, their participation in outdoor activities such as hiking and gardening, and other factors that would influence their exposure to tick habitats. Both the Kansas Extension professionals’ and Kansas community surveys were pilot tested with ten Extension professionals and ten Kansas community members, respectively, to improve clarity. The final versions included 20 items each. The Extension survey was open from 9 October to 9 November 2024, while the community survey ran from 15 January to 25 March 2025. Survey invitations for Extension professionals were distributed via email to relevant listservs, Extension newsletters, and during the annual conference. Community survey invitations were shared during listening sessions across Kansas, through local news reports, the Kansas Extension website, and flyers distributed by Family and Consumer Science Extension professionals.

Due to concerns about potential fraud, the initial Kansas community survey was closed after 20 days. A new Kansas community survey was launched with a new weblink and QR code on the 10th of February and closed on the 25th of March. The new survey incorporated enhanced fraud detection measures, including IP address tracking, bot detection, reCAPTCHA, RelevantID, and restrictions on multiple submissions. IP address tracking enabled us to filter out responses from individuals outside the state of Kansas. Bot detection algorithms helped identify and flag automated bot submissions that could skew the data. The inclusion of a reCAPTCHA question at the beginning of the survey ensured that only human respondents participated. RelevantID was used to analyze respondent metadata, allowing us to detect potential instances of multiple submissions from the same individual. Finally, the prevention of multiple submissions feature ensured that each participant could complete the survey only once, safeguarding against duplicate or inflated data [28]. Once a participant commenced the survey, they had 72 h to complete the questions, and afterwards any partially completed surveys were saved and stored within the survey data.

**Figure 1:**
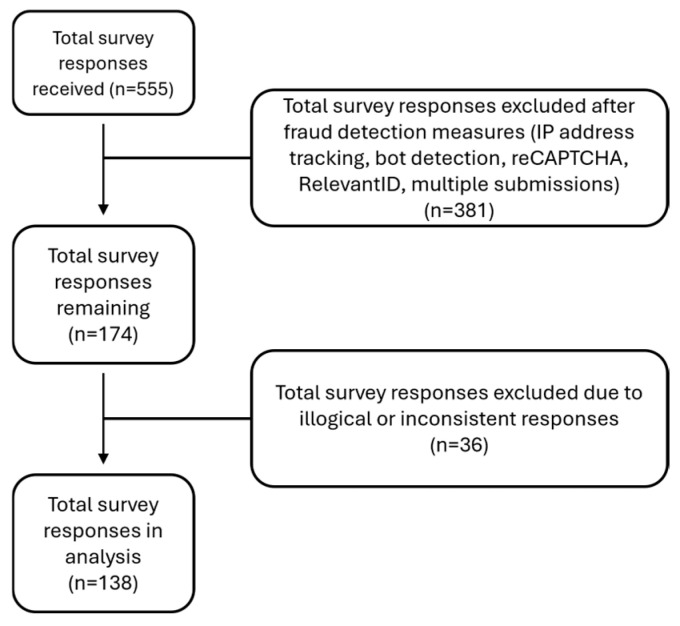
Flow diagram of survey response exclusions and final analytic sample.

A total of 555 community surveys were received; of these, 381 were excluded after fraud detection measures listed above. An additional 36 surveys were excluded due to illogical or inconsistent responses (e.g., answers that did not match the survey question). The final sample consisted of 138 community surveys (Figure 1).

### 2.3. Data Analysis

The survey responses were exported from Qualtrics (Qualtrics XM, Provo, UT, USA) to Microsoft Excel (2021, Microsoft Corp., Redmond, WA, USA) and subsequently analyzed using SPSS Statistics (version 29.0, IBM, Armonk, NY, USA). Participant demographic characteristics were analyzed descriptively. Age data was collected in categories for consistency with how Extension programs report demographic information to the U.S. Department of Agriculture. As a result, estimated means for age were calculated using weighted average of the midpoints of each grouped category using the following mid points: 23.5, 38.5, 53.5, and 65.5 for the matching age categories.

Categorical variables are presented as frequency (*n*) and percentage (%). Binary logistic regression was conducted to examine whether pet ownership, time spent on outdoor activities, age, and gender predicted the likelihood of having AGS. Pet ownership and time spent on outdoor activities were the primary predictors of interest, while age and gender were included as covariates. Age was included as a categorical variable with four levels, with age group 60+ serving as the reference category, while gender was coded as 1 = male and 2 = female. The dependent variable (self-reported AGS status) was coded as 0 = for individuals who did not have AGS and 1 = for those who self-reported having AGS. Odds ratios (ORs) and 95% confidence intervals (CIs) were reported with statistical significance set at *p* < 0.05.

## 3. Results

### 3.1. Participant Characteristics

#### 3.1.1. Extension Professionals

Of the 174 responses received for the Extension professionals’ survey, 144 were retained for analysis after excluding incomplete submissions, specifically those in which only the initial characteristic questions were answered. The majority of respondents were female (72%) and non-Hispanic White (93%), with 41% between the ages of 30 and 47 (Table 1). About 5% reported having AGS. Most participants reported serving in the Northeastern (28%) and Southeastern (25%) regions of Kansas.

#### 3.1.2. Kansas Community Participants

The initial and second Kansas community surveys received 178 and 377 responses, respectively, totaling 555 submissions. After eliminating fraudulent submissions, 138 valid responses (25%) remained for analysis. The majority of participants were female (63%), non-Hispanic/Latino (89%), white/Caucasian (90%), and aged 60 years or older (38%) (Table 1). Most respondents (42%) were from counties in Eastern Kansas particularly Reno, Wyandotte, Sedgwick and Crawford. Occupation-wise, participants were primarily retired (21%), teachers (8%) and business owners (6%). About 56% reported having AGS. In terms of lifestyle, 79% of participants owned pets, and the majority (33%) spent less than 5 h per week engaged in outdoor activities, such as gardening and yard work (35%), or adventure and nature exploration, including hiking, camping, birding, and mountaineering (30%). Unadjusted logistic regression analysis showed that participants with pets had significantly higher odds of having AGS (OR = 3.43, 95% CI (1.42, 8.32), *p* = 0.006), similarly, participants who spent more than ten hours outdoors per week also had increased odds of AGS (OR = 2.39, 95% CI (0.97, 5.90), *p* = 0.060), although this association did not reach statistical significance (Table 2). After adjusting for age and gender, the association between pet ownership and AGS status remained statistically significant and slightly stronger (OR = 4.04, 95% CI (1.57,10.41), *p* = 0.004). In contrast, the association between time spent on outdoor activities and AGS status remained non-significant after adjustment (OR = 2.11, 95% CI (0.81, 5.49), *p* = 0.128) (Table 3).

### 3.2. Participants’ Knowledge and Awareness of AGS

#### 3.2.1. Extension Professionals

**Figure 2:**
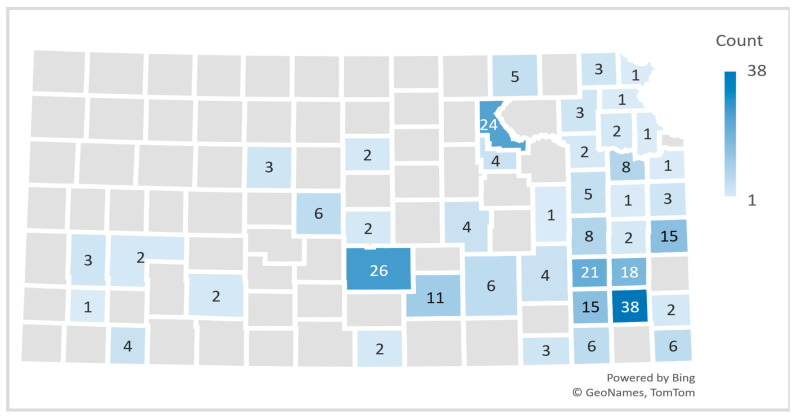
Geographic distribution of self-reported knowledge of AGS cases by Extension Professionals across Kansas counties.

The majority of Extension professionals who participated in the study were familiar with AGS, with 91% reporting awareness of the condition. A significant proportion (88%) correctly identified tick bites as the cause of AGS (Table 4). However, only 46% of participants were familiar with the specific symptoms associated with the condition. Among those who were aware of AGS symptoms, the most commonly reported were nausea (73%), abdominal pain (64%), hives (62%), and diarrhea (61%). In terms of community awareness, 54% of participants knew someone within their county who had been diagnosed with AGS. The majority (51%) had learned about AGS through friends or extension programming activities (13%). Participants’ self-reported knowledge of cases of AGS in their respective counties was mapped to estimate the geographic distribution of AGS across Kansas counties. Reno and Neosho seemed to have the highest number of cases (Figure 2). The majority (82%) did not know anyone who had recovered from AGS. Most participants (65%) felt there was insufficient information for the public about AGS treatment and management, and only 3% had had their extension offices approached for information pertaining to AGS, while 80% expressed interest in attending listening sessions on AGS.

#### 3.2.2. Kansas Community Participants

**Figure 3:**
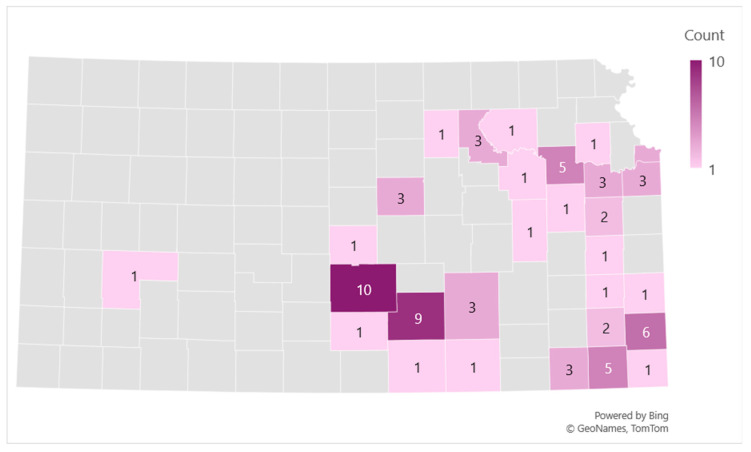
Geographic distribution of Community survey participants with self-reported AGS across Kansas counties.

A high percentage of Kansas community participants were familiar with AGS, with 95% aware of the condition, 96% understanding its cause and 88% recognizing its symptoms (Table 4). The most reported AGS symptoms were hives (67%) and abdominal pain (67%) followed by diarrhea (64%) and itching (64%). Furthermore, 84% of respondents knew someone diagnosed with AGS within their county, with 32% personally diagnosed and 28% learning about the condition through friends. Participants who self-reported as having AGS were mapped to estimate the geographic distribution of AGS across Kansas counties. Reno and Sedgwick counties seemed to have the highest number of cases (Figure 3). A significant proportion (76%) reported not knowing anyone who had recovered from AGS, while 14% knew an individual who had. Most participants (89%) felt there was insufficient public information about AGS treatment and long-term management, while 75% expressed interest in receiving AGS information.

### 3.3. Participants’ Perceived Impact of AGS on Individuals

#### 3.3.1. Dietary Restrictions

##### Extension Professionals

Extension professionals consistently emphasized the profound impact AGS has on the daily lives of those diagnosed. In particular, they highlighted the significant dietary and lifestyle adjustments required to manage the condition, with the strict dietary restrictions presenting a notable burden. Many individuals shifted to relying on poultry, fish, and vegetarian alternatives as primary sources of protein. Additionally, participants underscored the constant need for vigilance in avoiding less obvious sources of mammalian by-products such as gelatin, dairy, and certain sugars processed with animal-derived agents like bone char. Most individuals reported depending heavily on home-cooked meals, bringing their own food to social gatherings, and meticulously reading ingredient labels to prevent accidental exposure. One participant described their friends’ ordeal: “I met both individuals at a food preservation program that I did. They were both there because they wanted to learn more about how to preserve their own food because of the amounts of items in other foods that were causing symptoms. One was unable to have sugar due to the bone meal used during the milling process.” Another remarked about an individual they know: “They are pretty fatigued while they are transitioning to finding foods that they can handle eating. They are finding unique foods that are helping them that weren’t on their radar or mine before. They all seem to be very frustrated with having to alter their food habits.”

##### Kansas Community Participants

When asked about their perceptions of how AGS had impacted individuals they knew who had been diagnosed, respondents frequently emphasized the significant lifestyle and dietary challenges associated with the condition. A predominant concern centered on the strict dietary restrictions required to manage AGS, particularly the complete elimination of mammalian meat. Many also reported the necessity of avoiding dairy products and by-products, as well as limiting consumption or use of items containing alpha gal such as gelatin, carrageenan, and other hidden ingredients. In many cases, individuals had to adopt a fully vegan or plant-based diet and routinely scrutinize food labels to avoid inadvertent exposure to mammalian ingredients. One participant described the emotional toll of navigating this unfamiliar dietary landscape: “This was traumatizing, the first time I went grocery shopping, I sat on the floor in the store and cried because I couldn’t find food that I knew would be safe. Eventually I learned and so now I eat mostly chicken and turkey. I use almond milk, plant butter, and check ingredients of everything I buy,” (Table 5).

#### 3.3.2. Health Impact

##### Extension Professionals

Extension professionals also reported a range of adverse health complications associated with AGS, many of which significantly affect individuals’ quality of life. These included severe allergic reactions such as anaphylaxis, necessitating the regular use of antihistamines, epinephrine, or EpiPens. Respondents noted that individuals with AGS often had to alter medications prescribed for pre-existing conditions due to the presence of mammalian-derived ingredients. One participant shared, “They have had to change what medication they take and food they eat.” Another observed, “They also have to avoid glycerin in pills like Dayquil,” highlighting the hidden risks in common medications. Beyond physical health, participants also described the toll AGS takes on emotional and mental well-being. Fatigue, chronic stress, and anxiety were commonly reported, stemming from the constant vigilance required to avoid exposure and manage symptoms. Some individuals were described as having lost a significant amount of weight due to the strict dietary limitations and the reduced variety of safe food options.

##### Kansas Community Participants

Health-related complications were also noted. Several respondents described adverse reactions to medications containing mammalian-derived components such as gelatin. Some indicated that AGS appeared to exacerbate or trigger additional health conditions and food allergies, such as nut allergies, while others expressed concerns about maintaining adequate nutritional intake given the restricted dietary options. One parent remarked that: “It has definitely been a challenge for my son. He is nine and struggles with not being able to eat what his friends and family do. It’s also hard to make sure he is eating enough”. Another participant with AGS reported that “even with restricting mammalian meat and dairy from my diet, I continue to have allergic reactions to other foods and fumes. My doctor suspects that I have Mast Cell Activation syndrome that was brought on from having Alpha-Gal syndrome”.

#### 3.3.3. Social and Financial Impact

##### Extension Professionals

Extension professionals reported that individuals living with AGS often turn to home cooking as a primary strategy for managing their condition. Preparing meals at home allows for greater control over ingredients and reduces the risk of accidental exposure to mammalian products. However, this shift also comes with significant lifestyle challenges. Respondents noted the difficulty individuals face in attending social events, dining out, or participating in communal meals, experiences that are often accompanied by stress, anxiety, and emotional fatigue due to limited safe food and product options. This condition impacts not just individuals, but their families and friends. Participants highlighted how family members and friends often need to adapt their own habits, creating a ripple effect of lifestyle changes. This transition can be particularly difficult for individuals who previously consumed meat regularly, with some respondents describing the emotional strain of giving up familiar and culturally significant foods.

In addition to personal challenges, Extension professionals also noted the financial and professional consequences of AGS for those involved in livestock production, particularly beef producers. Several reported that individuals diagnosed with AGS were forced to modify or completely shift their farming operations to reduce exposure risks. One participant shared, “One friend raises cattle, they still do but have added emu to their operation”. Another wrote, “As a beef producer, one was devastated”. Others emphasized the broader cultural and economic impact, stating, “It was hard for everyone I know, especially since beef is such a large part of most Kansas diets”.

##### Kansas Community Participants

Social and emotional challenges were also widely reported. Participants highlighted difficulties attending social gatherings, including family events, potlucks, and meals at restaurants where mammalian meat was present or cross-contamination was possible. Many individuals resorted to bringing their own food to such occasions and described experiencing heightened anxiety and emotional distress during meals. One respondent described experiencing “high anxiety over eating at restaurants or any sort of shared meals, frustration over high amounts of hidden mammal byproducts in many foods and people’s lack of understanding”. The need to request ingredient lists and special accommodations often led to discomfort, and fear of accidental exposure contributed to social withdrawal in some cases. In addition, respondents reported strain on interpersonal relationships due to the increased planning and accommodation required to safely include individuals with AGS in shared meals or events.

Beyond social concerns, participants identified broader lifestyle implications. The need to find suitable alternatives for everyday products containing mammalian-based ingredients such as medications, cosmetics, and personal care items including shampoos and lotions added further complexity to daily life. Financial burden was another recurring theme. Respondents noted increased costs associated with purchasing specialty foods, acquiring separate kitchen appliances and utensils to avoid cross-contamination, and investing in tools such as food-scanning applications, EpiPens, and medications, some of which were not covered by insurance. One respondent succinctly summarized this burden, stating, “Food is more expensive and harder to find”. Another participant expressed frustration with losing access to medication that had been helpful: “I was on Dupixent, it helped, but insurance no longer covers it”. One individual reported, “I purchased entirely new dishes, pots and pans, silverware, etc., for my kitchen to avoid the possibilities of cross-contamination”. Similarly, the importance of readiness for allergic reactions was emphasized by another respondent: “I always have an EpiPen at home and at work, and I sometimes take it with me if dining somewhere new or travelling”.

### 3.4. Perceived Knowledge Gaps and Their Impact on AGS Management

When participants were asked about the adequacy of publicly available information regarding the treatment and management of AGS, the majority expressed dissatisfaction. Respondents consistently reported widespread gaps in awareness not only among the general public but also among healthcare professionals, the restaurant industry, and insurance providers. These knowledge deficits were seen as significant barriers to timely diagnosis, effective treatment, and daily management of the condition.

A recurrent theme was the difficulty in obtaining a correct diagnosis. Several individuals described delayed or missed diagnoses, sometimes spanning years, which they attributed to limited healthcare provider knowledge and the tendency to dismiss or minimize patient-reported symptoms. One participant stated, “There is very little that doctors know about it,” while another elaborated, “One of the greatest obstacles I face is that doctors don’t believe me when I tell them, they don’t understand how to treat me, and many of them seem to lack an understanding of what a mammal is.” Even individuals with a known family history of AGS described delays in obtaining a diagnosis, “There’s so little information that it took nearly 3 years to diagnose me even though a relative had it”.

Participants frequently emphasized the lack of training among healthcare providers and called for more widespread education. This concern extended to access to appropriate diagnostic tests and preventive interventions such as EpiPens. As some noted: “Doctors need to be educated! There needs to be access to the test!” and “there needs to be more doctor education on this and less shock about it. Easier diagnosis and preventative measures covered like EpiPens”.

Experiences with insurance companies also reflected systemic knowledge gaps. Respondents reported denials of coverage for testing, treatment, and emergency medications due to lack of recognition of AGS as a legitimate medical condition. One participant recounted, “I have it. Was treated with lots of skepticism after a full anaphylactic emergency room visit. I figured it out and requested the lab for verification and was told insurance probably wouldn’t cover it. That changed 3 weeks later when the result came in. Had an EpiPen prescription for those 3 weeks but insurance wouldn’t cover it”.

Despite receiving care from allergists, many respondents described treatment plans that were largely limited to basic antihistamines. As one individual reported, “No, I have not seen any information on how to treat or manage AGS. My allergist only has me taking an allergy pill each day”.

Many individuals reported turning to online sources for information and support. Community-level educational resources were described as nearly nonexistent. “I do not know of any educational resources in my community. My information is from researching the condition online,” said one participant. Others highlighted the mixed quality of online content: “I believe that there is much misinformation mixed with good information on social media sites, but reputable sources lack good information on specific aspects of food manufacturing”.

Participants also noted a lack of social support and general recognition of AGS as a serious, potentially life-threatening condition. “I wish people accepted allergies as life-threatening conditions”, one respondent remarked. The lack of awareness in the restaurant industry was also emphasized as a practical concern, with one participant explaining, “I think more people should be aware, especially in the restaurant industry. We are able to go to a select few due to our own research”.

While most experiences pointed to a general deficit in healthcare preparedness, a few respondents reported isolated positive encounters, such as, “Many including medical personnel know little about Alpha-gal. I was fortunate with having a nurse practitioner that immediately tested me for tick-related diseases”.

## 4. Discussion

This study aimed to assess public awareness and understanding of Alpha-Gal Syndrome (AGS), an allergic reaction to galactose-alpha-1,3-galactose, as well as knowledge of its symptoms and available management resources. Additionally, it examined the distribution of AGS based on self-reports, its impact, and the challenges associated with long-term symptom management in rural communities across Kansas. The study reveals awareness of AGS among both Kansas community members and Extension professionals but also significant dietary, social, financial, and healthcare-related challenges for individuals living with the condition. Moreover, our results highlight critical gaps in healthcare system preparedness and support infrastructure for managing AGS across rural Kansas communities.

Among both groups of participants, awareness of AGS was significantly high, with over 90% of respondents demonstrating knowledge of the condition and its tick-borne etiology. This may be reflective of the increasing prevalence of AGS in Eastern Kansas and the strong word-of-mouth communication within communities, given 84% of community members reported knowing someone within their county who had been diagnosed. Extension professionals reported slightly lower awareness of symptoms, with only 46% able to accurately identify key symptoms of AGS. This disparity may necessitate further professional development for Extension professionals to support their role as community health and wellness educators and communicators.

Geographic distribution data revealed that AGS cases were concentrated in Reno, Neosho, and Sedgwick counties. Notably, both Reno and Neosho counties were also identified by the Kansas Department of Health and Environment (KDHE) as having a high number of suspected cases, exceeding 87 cases per million population annually between 2017 and 2022 [29]. Thompson et al. [25] further noted that the geographic distribution of AGS mirrors that of ehrlichiosis, a tick-borne illness caused by bacteria *Ehrlichia chaffeensis* and *E. ewingii*, which are also transmitted by the lone star tick. This relationship is supported by our findings and is consistent with KDHE data on ehrlichiosis and lone star tick distribution across Kansas [30]. This suggests these areas may warrant targeted public health interventions.

The results suggest that pet ownership is significantly associated with increased odds of AGS, with an unadjusted odds ratio (OR) of 3.43 and an adjusted OR of 4.04 after controlling for age and gender. This increase in OR after adjustment suggests that age and gender had a negative confounding effect on the association, given our sample’s demographic imbalance, with females comprising 63% and 38% of participants aged 60+. This pattern aligns with findings from Ross et al. [31], who reported a higher national prevalence of AGS among males than females and an increased likelihood of a positive AGS test with age, particularly after age 40. The wide confidence interval reflects limited precision in the effect estimate. This highlights the need for further studies with larger sample sizes to confirm the observed association. Existing literature on the relationship between pet exposure and AGS remains limited. Mateo Borrega et al. [32], in a study conducted in Spain, reported a significant correlation between pet exposure and the presence of alpha-gal-specific IgE antibodies, with the association being stronger among individuals exposed to dogs than to cats. Similarly, Chinuki et al. [33] observed that Japanese individuals with red meat allergy were more likely to own dogs. They hypothesized that dogs could act as vectors for tick transmission, potentially introducing ticks to individuals during activities such as walking dogs in grassy areas or petting them after outdoor exposure. In contrast, González-Quintela et al. [34] found a stronger association between alpha-gal sensitization and cat ownership in both Spanish and Danish cohorts. It has been hypothesized that airborne exposure to pet dander, particularly from cats, may increase allergic sensitization [35,36]. Despite these associations, the role of pet exposure in AGS development remains inconclusive. In a narrative review, Dr. Commins [37], a leading expert on AGS, argued that pet dander is unlikely to be a significant risk factor for reactive individuals. He emphasized that eating mammalian meat alone was not sufficient to trigger an immune response to alpha-gal, and that other forms of mammalian exposure such as pet dander, have not been conclusively linked to AGS development. Based on this, Commins does not recommend pet removal for AGS patients. Instead, he advises healthcare professionals informing patients that pet dander may contain alpha-gal and recommends washing pets monthly to minimize potential exposure [37].

Participants who spent more than ten hours outdoors per week had higher odds of having AGS, but the odds slightly decreased after adjusting for age and gender from 2.39 to 2.11, and the association remained statistically non-significant. The slight decrease in the OR suggests both gender and age may have had a small confounding effect, given our sample composition. Although this association did not reach statistical significance, the observed positive trend suggests a potential dose–response relationship. Notably, the wide confidence interval reflects limited precision in the effect estimate, which highlights the need for further studies with larger sample sizes to confirm this association. Our research findings are consistent with previous research showing that increased participation in outdoor activities is associated with increased tick bites, a key factor in the pathogenesis of AGS [32]. Commins [37] recommends that AGS patients take precautions to prevent tick exposure, as repeat bites may boost alpha-gal-specific IgE levels. Supporting this, Kim et al. [38] found that IgE levels declined among individuals who reduced their exposure to tick bites, although the rate and extent of decline varied. Iweala et al. [39] suggest that this variability may be influenced by factors such as dietary intake of mammalian meat and co-factors including medications, alcohol, physical activity, and continued tick exposure. Notably, Commins [40] reported that in some cases, alpha-gal-specific IgE levels decreased to a point where patients were able to gradually reintroduce mammalian meat into their diets.

Among both community participants and Extension professionals, the most profound effects of AGS were dietary/nutritional, emotional and social in nature. Respondents described the necessity of making significant dietary changes, with a significant number adopting vegetarian or plant-based diets to better manage their symptoms. However, research indicates that many health professionals are not adequately equipped to guide individuals through the complexities of a plant-based lifestyle without compromising essential nutritional and health needs [41]. As with other food allergies, managing AGS presents several challenges. These include the potential for overly restrictive eating patterns, unintentional exposure to allergens through cross-contamination, limited knowledge about which foods to avoid, inadequate allergen labeling on products, difficulties in identifying trigger foods and safe portion sizes. Collectively, these factors can have long-term consequences for an individual’s overall health and quality of life [42].

Avoidance diets may lead to nutritional deficiencies if not carefully managed with the support of a dietitian [43,44]. Eliminating red meat requires careful substitution with other protein-rich foods like poultry, fish, legumes, and soy products. Vaz-Rodrigues [45] suggests that individuals diagnosed with AGS should collaborate with a dietitian knowledgeable about the condition to monitor their iron and vitamin B12 levels. The dietitian should also evaluate the need for supplementation, particularly if the individual is allergic to gelatin and milk [20,46]. According to Mofidi [43], individuals with AGS who must eliminate key food groups, such as milk, should find alternative sources of calcium, vitamin D, and B vitamins, with fortified soy milk being a viable substitute [44]. Education on label reading, allergen identification, and suitable food alternatives remains critical to ensure both safety and nutritional adequacy [47].

The social impact of AGS was well reported with respondents describing the anxiety and emotional exhaustion related to social interactions, fear of accidental exposure and strained interpersonal relationships. Financial strain also emerged as a prominent theme, encompassing the cost of specialty foods, new kitchenware, and uncovered medical expenses such as AGS testing and treatment. For individuals whose livelihood revolved around beef production, AGS not only posed personal health risks but also professional and economic disruption, an angle seldom reported in AGS literature yet vital in agricultural states such as Kansas. Collectively, these accounts illustrate the “invisible burden” of AGS and how it affects the physical health of those diagnosed as well as their emotional resilience, daily routines, and overall quality of life.

Despite high community awareness, participants consistently expressed their frustration with the lack of accessible, reliable information, particularly around treatment, symptom management, and safe food sourcing. A recurring concern was the limited knowledge among healthcare providers. Carpenter and associates [24] found that in a web-based survey of 1500 healthcare professionals (including primary care physicians, pediatricians, physician assistants, and nurse practitioners), 42% had never heard of AGS, and among those who had, nearly half (48%) were unaware of the appropriate diagnostic tests. The majority expressed interest in clinical resources, including diagnostic and management guidelines. Similar findings have been reported in smaller studies [48,49], reinforcing the urgent need for improved professional education.

Participants also reported delayed diagnoses, disbelief from clinicians, and a lack of individualized care plans, resulting in prolonged suffering, increased healthcare use, and likely underreporting of AGS cases. These findings highlight the critical need for targeted public health campaigns, ongoing professional development for healthcare and community educators (Extension professionals), and formal recognition of AGS in clinical guidelines and insurance policies. Additionally, there is also a need for community-level education and standardized protocols for diagnosis and long-term care.

The results of this study can aid in initiating national surveillance efforts for this emerging allergic condition and guide geographically focused outreach to high-risk communities. Whether the increasing numbers of suspected AGS cases reflect increased awareness, increasing emergence, or both remains unclear. However, the responses highlight the urgent need for improved education among healthcare professionals, public awareness campaigns, and the development of accessible, evidence-based resources to support individuals with AGS and their families. Integrating AGS awareness into existing tick-borne disease prevention efforts, especially in counties determined to have high prevalence, will facilitate early detection and reduce disease burden. Future research should investigate long-term outcomes for AGS patients, diagnostic barriers in underserved communities, and interventions that address their challenges.

## 5. Limitations

A significant limitation of this study was the presence of a substantial number of fraudulent responses during the data collection phase. While rigorous data cleaning procedures were applied to improve the validity of the dataset, the high volume of unusable responses may reflect vulnerabilities in the survey distribution method, particularly in online environments where open-access links can be exploited by bots or individuals seeking incentives. The exclusion of these responses, although necessary for data integrity, reduced the effective sample size and may have influenced the demographic balance within the final dataset. Consequently, these factors may limit the generalizability of the findings and underscore the importance of implementing stronger fraud-prevention mechanisms in future studies.

Additionally, self-selection bias is an important consideration, given a significant portion of participants self-reported having AGS, due to the voluntary nature of participation. Individuals affected by AGS may have been motivated to complete the survey, which may have inflated the observed associations. In the absence of confirmed clinical diagnoses, reliance on self-reported symptoms may have led to overestimation of AGS distribution estimates. Furthermore, because age data was collected using grouped categories, estimated means for age were reported rather than the true means. In addition, there was an imbalance in the age range distribution across groups. Most Extension professionals were aged 30–47 years, whereas most community participants were 60 years or older. These differences likely reflect the demographic profile of Extension programming, which tends to engage a larger proportion of adults aged 60 and above.

## 6. Conclusions

While AGS awareness is growing in Kansas, individuals living with the condition continue to face significant dietary, emotional, financial, and systemic challenges. The data reveals a critical gap between awareness and action, that is, between knowing about AGS and being equipped to live with it safely and sustainably. Addressing this gap will require coordinated efforts across public health, medical, agricultural, and educational sectors to ensure that no Kansan navigating AGS is left unsupported.

## Figures and Tables

**Table 1:** Characteristics of Extension Professionals and Kansas Community participants.

	Extension Professionals (*n* = 144)	Community Participants (*n* = 138)
Characteristic	*n* (%)	*n* (%)
Age		
18–29 years	35 (24)	15 (11)
30–47 years	59 (41)	43 (31)
48–59 years	29 (20)	28 (20)
60+ years	19 (13)	52 (38)
Choose not to provide	2 (1)	-
Estimated mean age	41.5	50.1
Gender		
Female	104 (72)	87 (63)
Male	37 (26)	50 (36)
Choose not to provide	3 (2)	1 (1)
Race		
White or Caucasian	134 (93)	124 (90)
Black or African American	-	9 (7)
American Indian/Alaska Native	2 (1)	2 (1)
Asian	2 (1)	1 (1)
Native Hawaiian/Pacific islander	-	1 (1)
Two or more races	3 (2)	-
Choose not to provide	3 (2)	1 (1)
Ethnicity		
Non-Latino/Hispanic	133 (93)	115 (89)
Latino/Hispanic	4 (3)	6 (5)
Choose not to provide	6 (4)	9 (7)
AGS status		
Currently has AGS	7 (5)	75 (56)
Doesn’t have AGS	117 (87)	53 (40)
Doesn’t know	10 (8)	6 (5)
Pet ownership		
Has at least one pet	-	105 (79)
Doesn’t have pets	-	28 (21)

Note: Percentages are rounded to the nearest whole number and may not total 100% due to rounding. Calculations are based on the number of valid (non-missing) responses. Estimated means for age were calculated using a weighted average of the midpoints of each grouped category (mid points used: 23.5, 38.5, 53.5, 65.5), since age data was collected as categories.

**Table 2:** Unadjusted Logistic Regression models predicting the likelihood of AGS from pet ownership and time spent outdoors (separate models).

Predictor	B	Wald	*p*	OR (Exp (B))	95% CI for OR
Model 1: Pet ownership					
Pet ownership	1.23	7.44	0.006	3.43	(1.42, 8.32)
Model 2: Time outdoors					
Time spent outdoors	0.87	3.54	0.060	2.39	(0.97, 5.90)

Note: The dependent variable is AGS status coded 0 = do not have AGS, and 1 = have AGS; pet ownership coded: 1 = yes, 0 = No; Time spent outdoors coded: 1 = >10 h, 0 = ≤10 h per week. OR = odds ratio, CI = confidence interval.

**Table 3:** Adjusted Logistic Regression models predicting the likelihood of AGS based on pet ownership and time spent outdoors, controlling for age and gender.

Predictor	B (Pet Model)	OR (95% CI) (Pet Model)	*p* (Pet Model)	B (Outdoor Model)	OR (95% CI) (Outdoor Model)	*p* (Outdoor Model)
Pet ownership	1.40	4.04 (1.57, 10.41)	0.004	-	-	-
Time spent Outdoors	-	-	-	0.74	2.11 (0.81, 5.49)	0.128
Age (1)	0.54	1.72 (0.49, 6.05)	0.399	−0.16	0.86 (0.18, 4.11)	0.845
Age (2)	0.51	1.67 (0.44, 6.36)	0.455	−0.26	0.77 (0.14, 4.25)	0.768
Age (3)	0.92	2.50 (0.72, 8.66)	0.147	−0.39	0.67 (0.14, 3.18)	0.618
Gender (1)	−0.73	0.48 (0.22, 1.05)	0.065	−0.67	0.51 (0.19, 1.32)	0.165

Note: pet ownership coded: 1 = yes, 0 = No; Time spent outdoors coded: 1 = >10 h, 0 = ≤10 h per week; gender coded: 1 = male, age coded: 1 = 18–29, 2 = 30–47, 3 = 48–59 years. OR = odds ratio, CI = confidence interval.

**Table 4:** Knowledge and perceptions of Extension Professionals and Kansas Community participants.

	Extension Professionals (*n* = 144)	Community Participants (*n* = 138)
Question	*n* (%)	*n* (%)
Knowledge of AGS		
Yes	127 (91)	130 (95)
No	13 (9)	7 (5)
Knowledge of AGS cause (tick bite)		
Yes	123 (88)	129 (96)
No	17 (12)	6 (4)
Familiarity with AGS symptoms		
Yes	64 (46)	120 (88)
No	59 (42)	15 (11)
Don’t know	17 (12)	1 (1)
Reported known symptoms *		
Abdominal pain	47 (64)	84 (67)
Hives	46 (62)	84 (67)
Diarrhea	45 (61)	80 (64)
Nausea	54 (73)	78 (63)
Vomiting	42 (57)	56 (45)
Heartburn or indigestion	17 (23)	48 (39)
Itching	33 (45)	79 (64)
Swelling of the lips	39 (53)	77 (62)
Shortness of breath	32 (43)	60 (48)
Cough	12 (16)	44 (35)
Wheezing	16 (22)	40 (32)
People you know are diagnosed with AGS		
Knows at least one person	67 (54)	106 (84)
How did you find out they had AGS		
Friends	32 (51)	31 (28)
Respondent has AGS	4 (6)	36 (32)
Family	6 (9)	25 (23)
Programming	8 (13)	-
Word of mouth	6 (10)	-
Church	1 (2)	4(4)
Social media	5 (8)	2 (2)
School	1 (2)	-
Work	-	6 (5)
The person with AGS told them	-	5 (5)
How many people do you know who have recovered from AGS		
None	50 (82)	83 (76)
One person	10 (16)	15 (14)
More than one person	1 (2)	12 (10)
Think that not enough information is available on AGS		
Yes	2 (2)	2 (2)
No	89 (65)	119 (89)
Not sure	45 (33)	13 (10)
Interest in receiving AGS information		
Yes	107 (80)	101 (75)
No	27 (20)	20 (15)
Not sure	-	13 (10)

Note: Percentages are rounded to the nearest whole number and may not total 100% due to rounding. Calculations are based on the number of valid (non-missing) responses. * Participants could select more than one option; therefore, percentages may total more than 100%.

**Table 5:** Illustrative participant quotes related to experiences with AGS.

Theme	Representative Quote
Emotional and Psychological impact	“This was traumatizing, the first time I went grocery shopping, I sat on the floor in the store and cried because I couldn’t find food that I knew would be safe.”
	“They all seem to be very frustrated with having to alter their food habits.”
	“High anxiety over eating at restaurants or any sort of shared meals, frustration over high amounts of hidden mammal byproducts in many foods and people’s lack of understanding.”
Dietary Challenges	“Eventually I learned and so now I eat mostly chicken and turkey. I use almond milk, plant butter, and check ingredients of everything I buy.”
	“They are finding unique foods that are helping them that weren’t on their radar or mine before.”
	“It has definitely been a challenge for my son. He is nine and struggles with not being able to eat what his friends and family do.”
Health impact and System barriers	“Even with restricting mammalian meat and dairy from my diet, I continue to have allergic reactions… My doctor suspects that I have Mast Cell Activation syndrome…”
	“I was on Dupixent, it helped, but insurance no longer covers it.”
	“There is very little that doctors know about it… doctors don’t believe me when I tell them… seem to lack an understanding of what a mammal is.”
	“Doctors need to be educated! There needs to be access to the test!”
	“No, I have not seen any information on how to treat or manage AGS.”
	“I believe that there is much misinformation… but reputable sources lack good information…”
	“I was treated with lots of skepticism after a full anaphylactic ER visit…”
	“I do not know of any educational resources in my community. My information is from researching the condition online.”
Social and Financial impact	“One friend raises cattle, they still do but have added emu to their operation.”
	“As a beef producer, one was devastated.”
	“It was hard for everyone I know, especially since beef is such a large part of most Kansas diets.”
	“Churches need more info on special diets especially alpha-gal-friendly diets.”
	“I think more people should be aware, especially in the restaurant industry.”
	“I purchased entirely new dishes, pots and pans, silverware, etc., for my kitchen to avoid the possibilities of cross-contamination.”
	“Food is more expensive and harder to find.”
	“I always have an EpiPen at home and at work… but insurance wouldn’t cover it.”

## Data Availability

Data supporting the reported results is available upon request due to privacy and ethical reasons.

## Abbreviations

The following abbreviations are used in this manuscript:

AGS	Alpha-gal syndrome
IgE	Immunoglobulin E
NSAIDS	Nonsteroidal anti-inflammatory drugs
KDHE	Kansas Department of Health and Environment
